# Supplementary figures and images for: Higher frequency of interstate over international transmission chains of SARS-CoV-2 virus at the Rio Grande do Sul - Brazil state borders
Source: Virus Res. 2024 Dec 17;351:199500. doi: 10.1016/j.virusres.2024.199500 (PMC11720880; doi:10.1016/j.virusres.2024.199500)

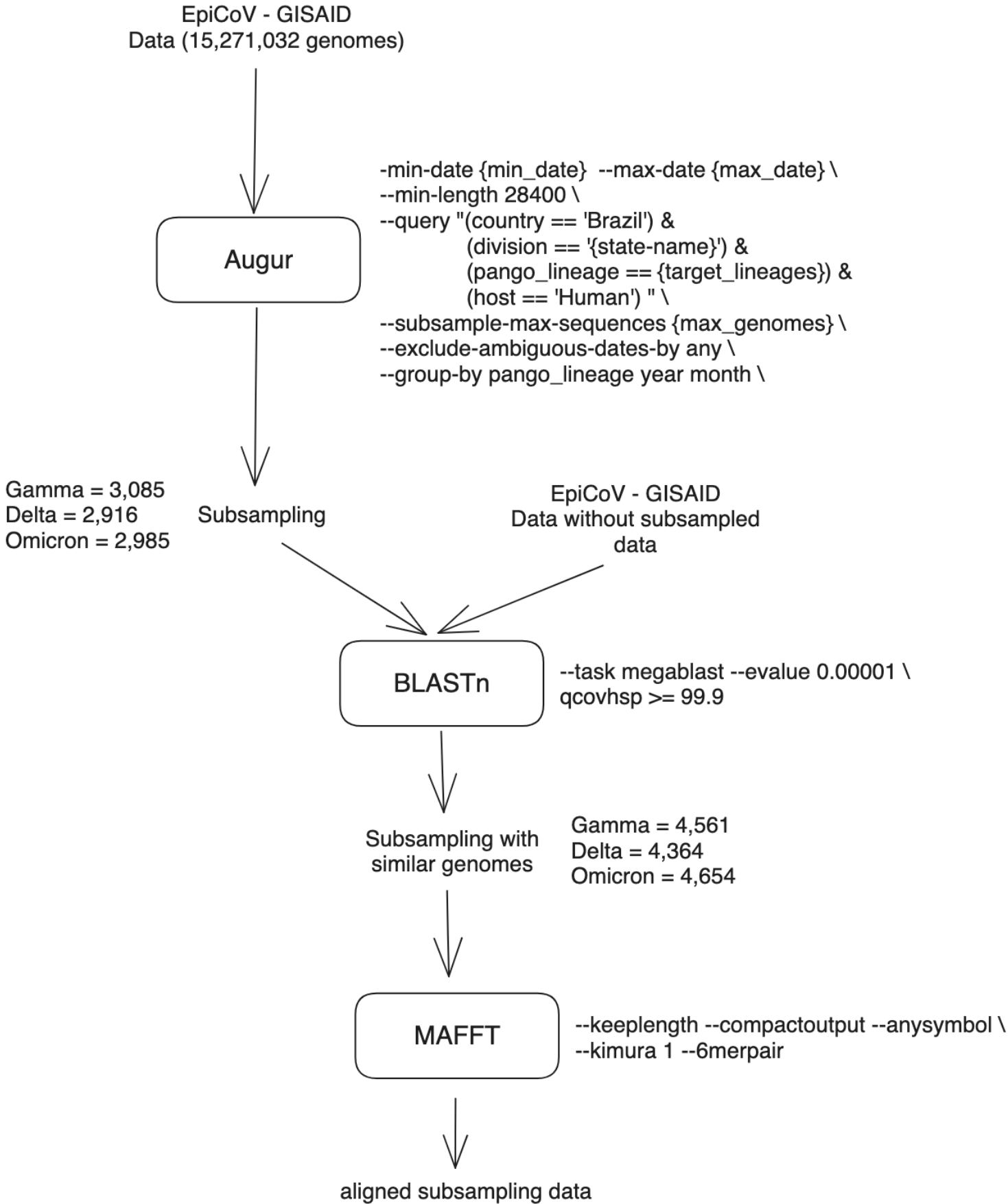

Supplement: Supplementary file 1 [file mmc1.pdf]

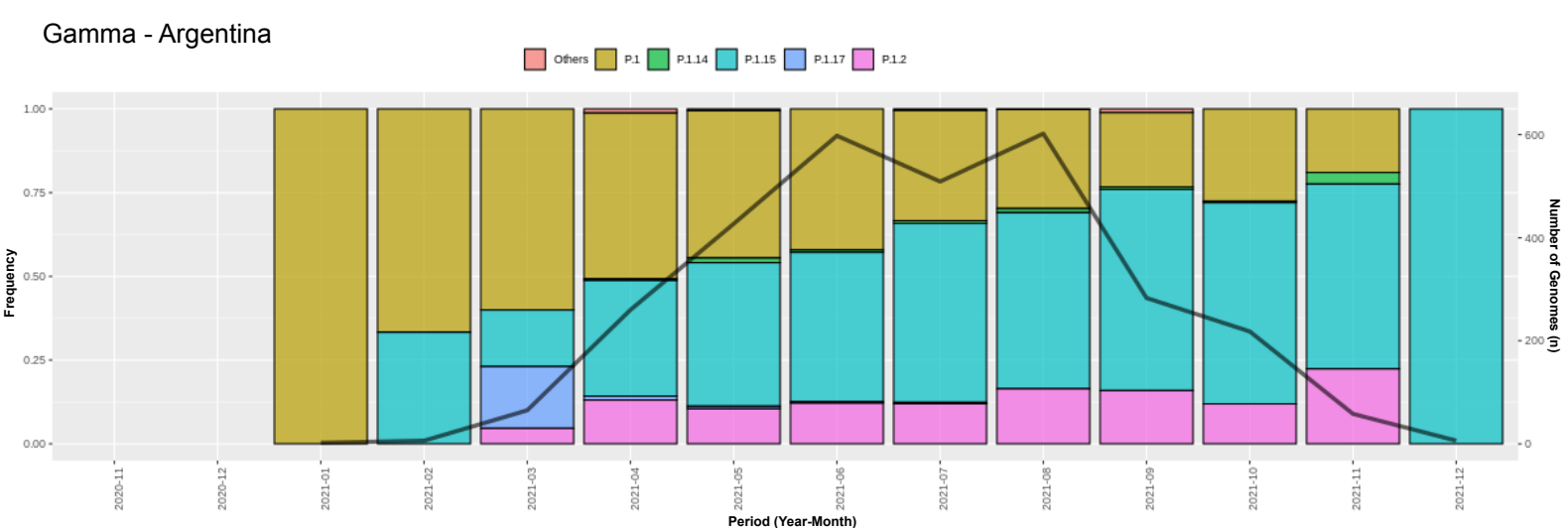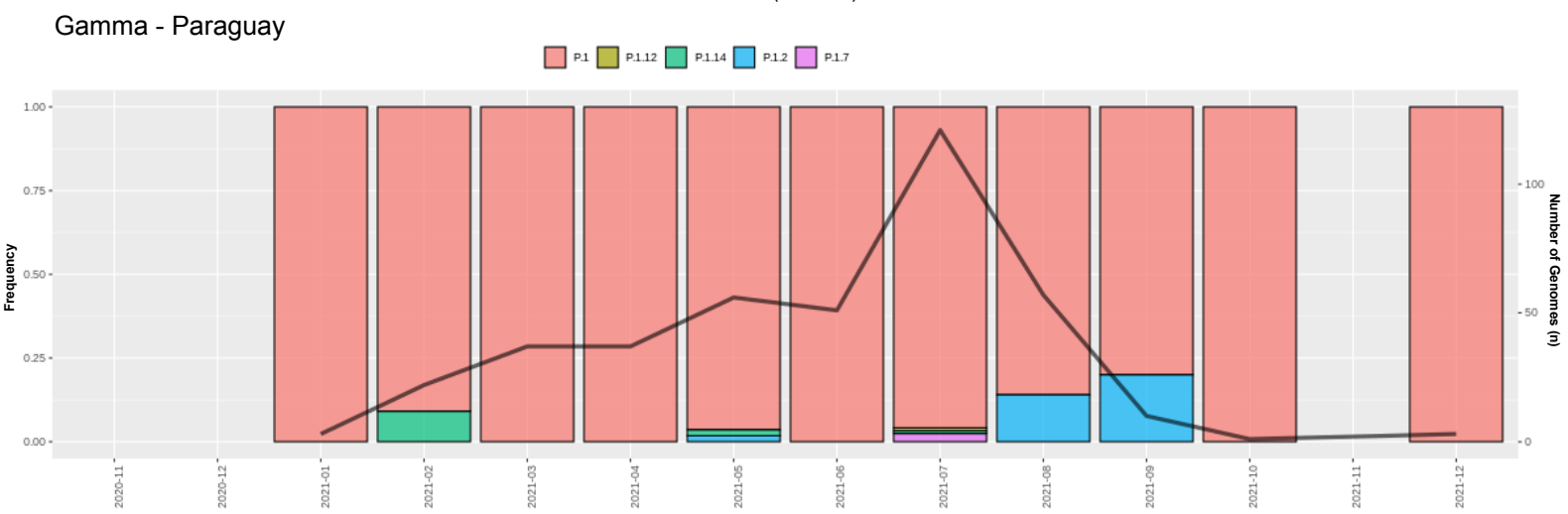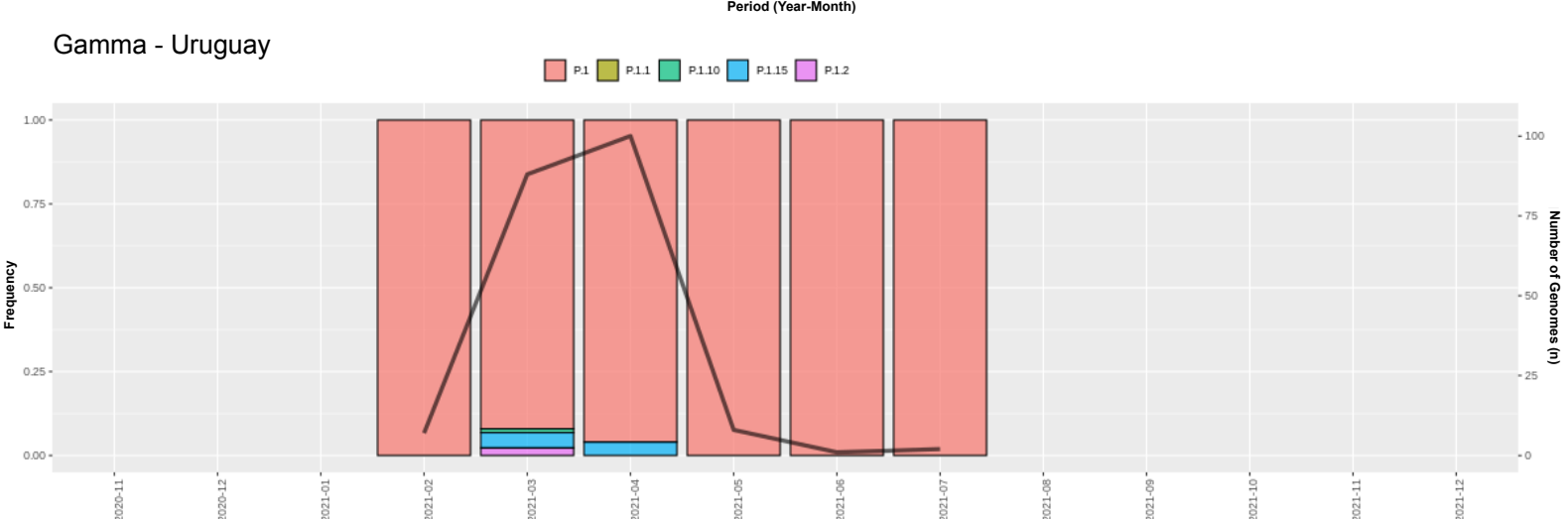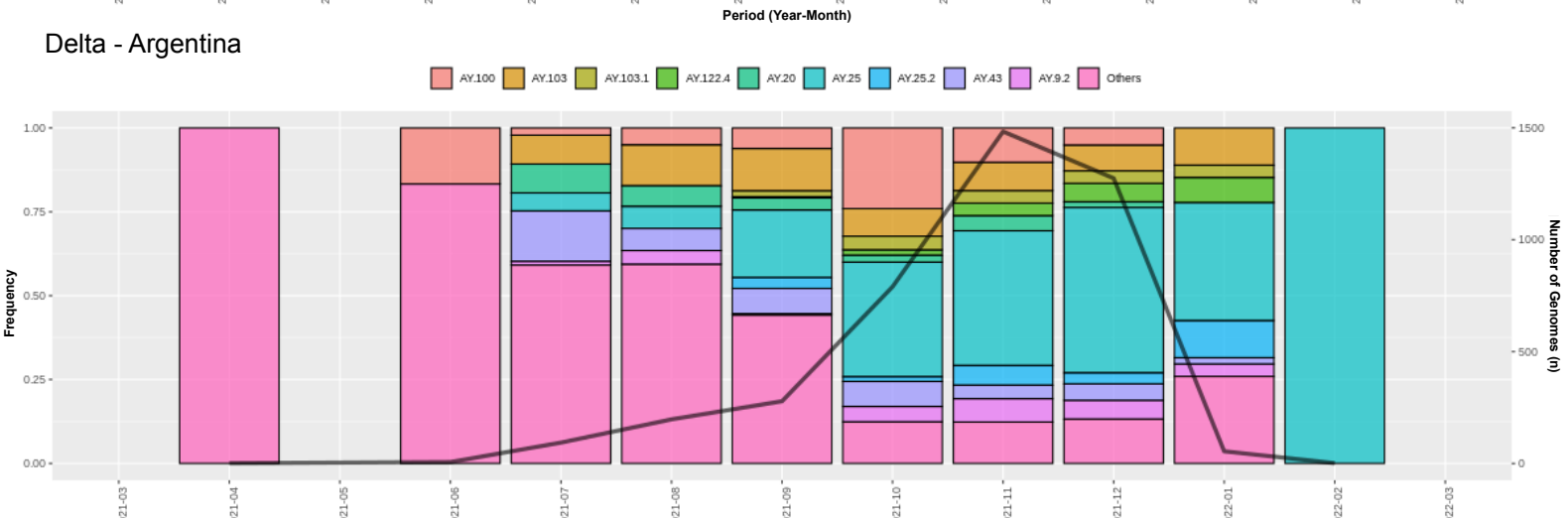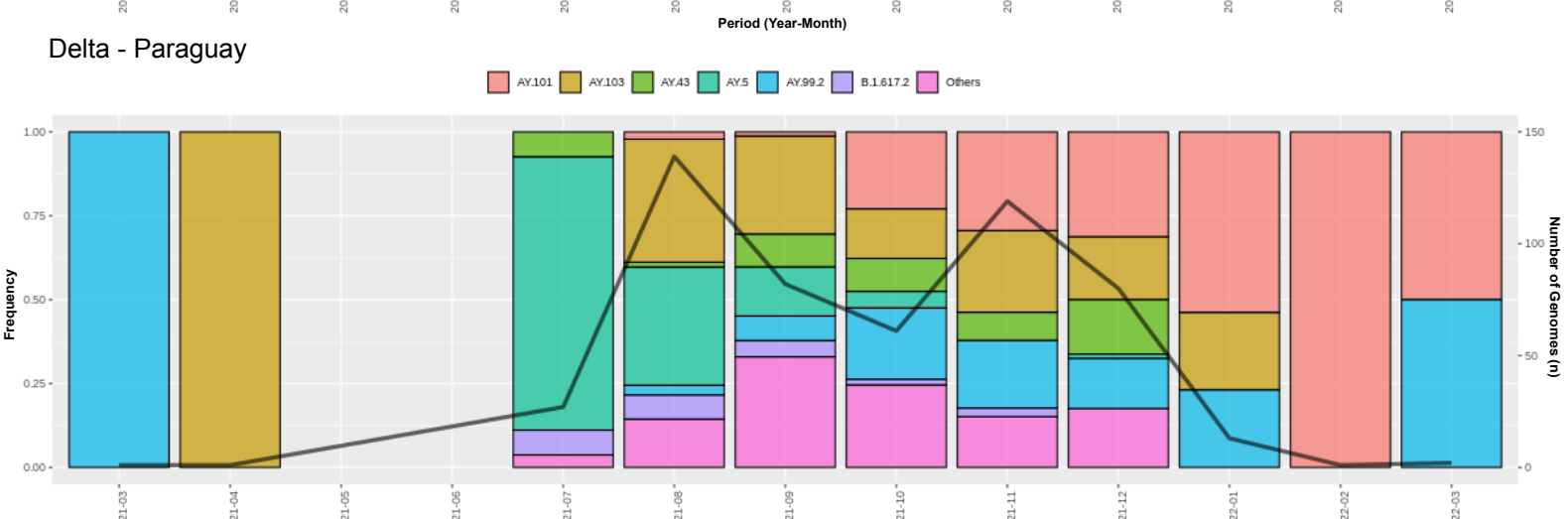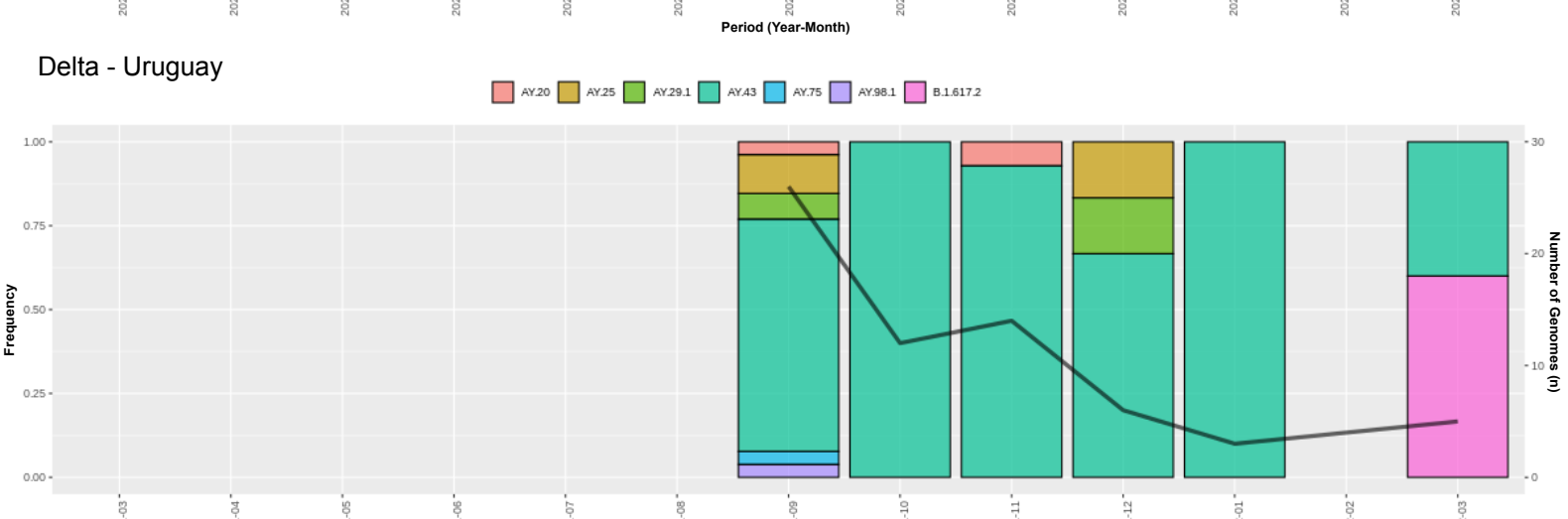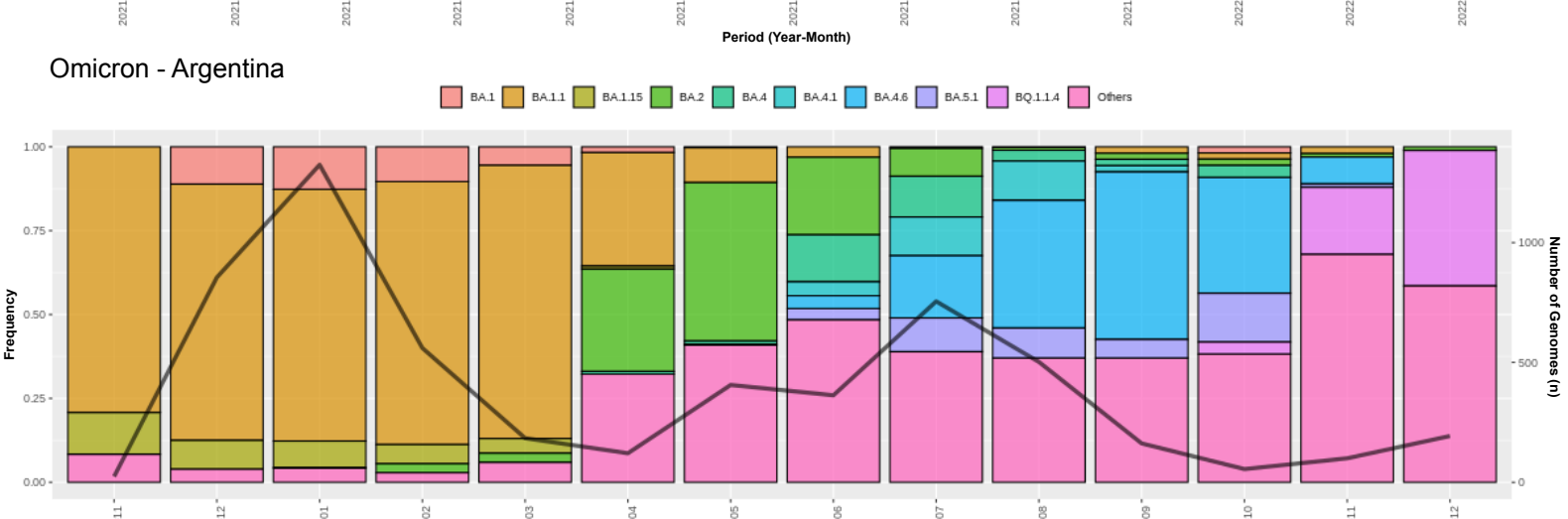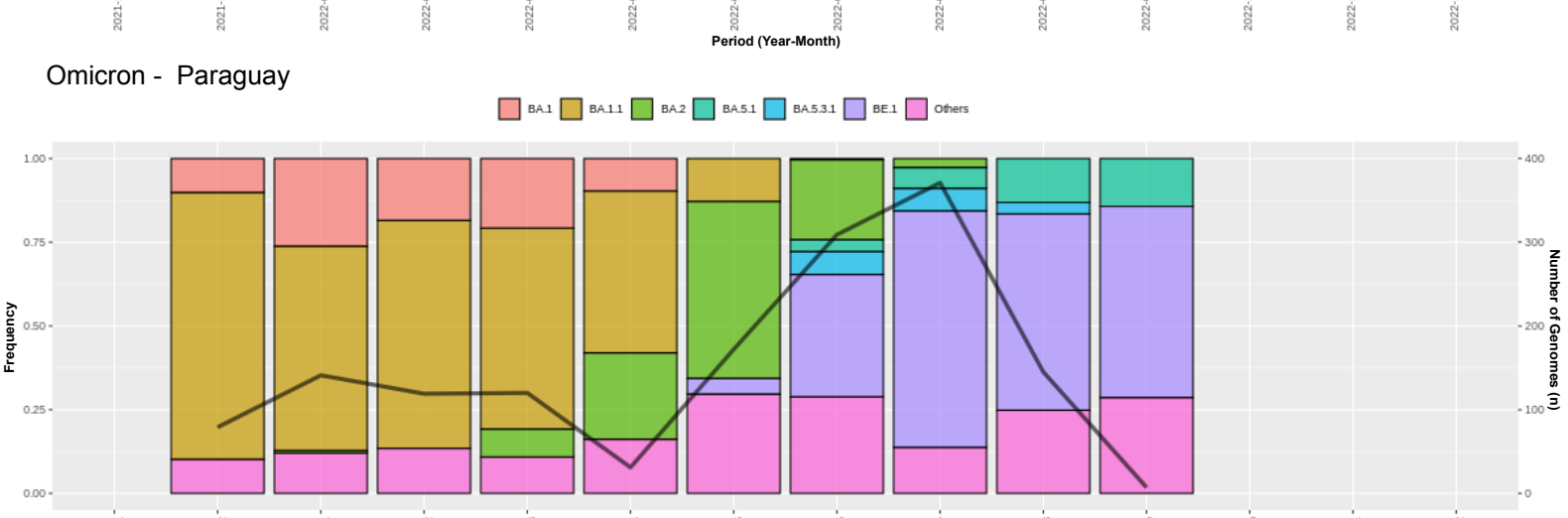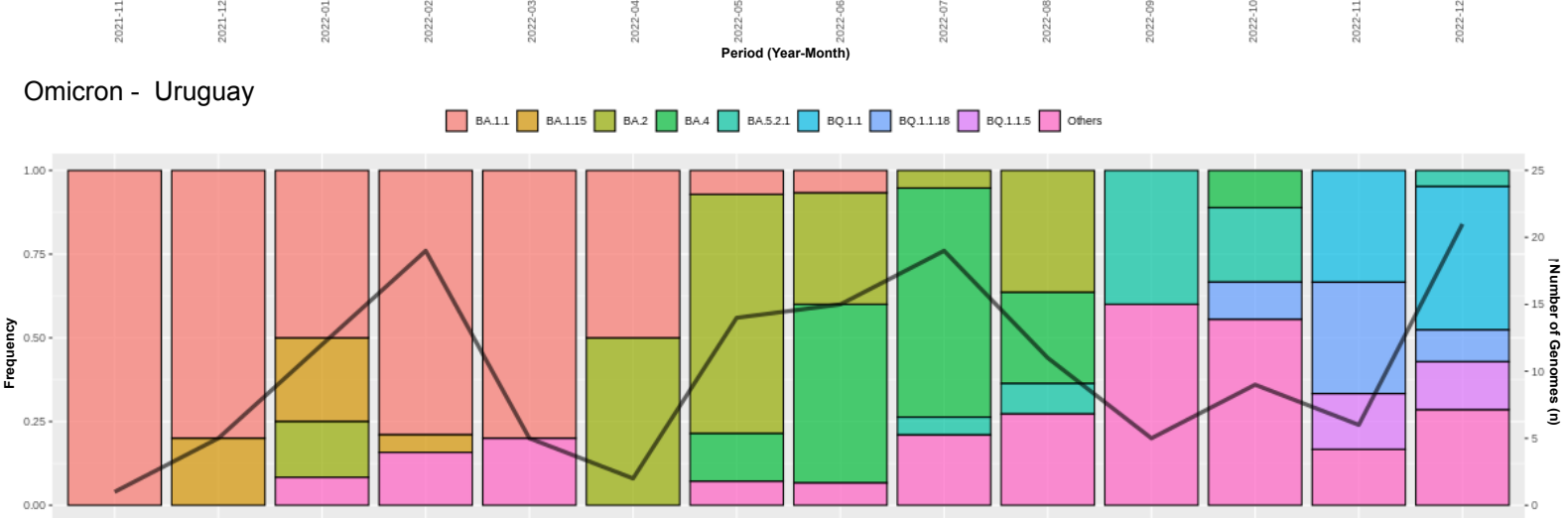

Supplement: Supplementary file 9 [file mmc9.pdf]
