# Supplementary material for: Higher frequency of interstate over international transmission chains of SARS-CoV-2 virus at the Rio Grande do Sul - Brazil state borders
Source: Virus Res. 2024 Dec 17;351:199500. doi: 10.1016/j.virusres.2024.199500 (PMC11720880; doi:10.1016/j.virusres.2024.199500)
Supplement: Supplementary file 2 [file mmc2.docx]

**SupplementaryData2:** Sequences on long branches that are removed from phylogenies, and their genome coverage breadth.

Sequences sequenced in this study

| sample (internal code) | coverage breadth (%) |
| --- | --- |
| IAM4930 | 99,99 |
| IAM12196 | 95,78 |
| IAM8761 | 94,94 |
| IAM8765 | 94,77 |
| IAM8794 | 94,7 |
| IAM8758 | 94,69 |
| IAM8798 | 94,48 |
| IAM8777 | 94,44 |
| IAM8795 | 94,39 |
| IAM8784 | 94,22 |
| IAM8773 | 94,09 |
| IAM8787 | 94,03 |
| IAM8793 | 94,03 |
| IAM8796 | 93,98 |
| IAM8790 | 93,96 |
| IAM8779 | 93,78 |
| IAM8792 | 93,78 |
| IAM8797 | 93,43 |
| IAM8775 | 92,11 |
| IAM8772 | 90,99 |
| IAM8786 | 89,98 |
| IAM4501 | 89,59 |
| IAM8764 | 85,25 |
| IAM5069 | 80,97 |
| IAM5156 | 79,64 |
| IAM5147 | 79,53 |
| IAM5148 | 79,49 |
| IAM5143 | 79,31 |
| IAM5140 | 78,99 |
| IAM5138 | 78,79 |
| IAM5102 | 78,76 |
| IAM5139 | 78,73 |
| IAM5136 | 78,64 |
| IAM5093 | 78,51 |
| IAM5132 | 78,23 |
| IAM5128 | 78,13 |
| IAM5098 | 78,11 |
| IAM5096 | 78,08 |
| IAM5074 | 78,07 |
| IAM5090 | 78,07 |
| IAM5126 | 78 |
| IAM5111 | 77,88 |
| IAM5124 | 77,86 |
| IAM5113 | 77,56 |
| IAM5116 | 77,33 |
| IAM5088 | 77,29 |
| IAM5100 | 76,54 |
| IAM5077 | 76,28 |
| IAM5097 | 75,55 |
| IAM5071 | 75,23 |
| IAM5064 | 75,14 |
| IAM8759 | 75,09 |
| IAM5070 | 74,07 |
| IAM12205 | 72,44 |
| IAM12206 | 72,12 |

Sequences from GISAID

**Gamma**

| sample | coverage breadth (%) |
| --- | --- |
| Brazil/PA-ITV-3109/2021 | 99,97 |
| Brazil/PI-FIOCRUZ-FioPi60/2021 | 99,70 |
| Brazil/RR-ITV-178708/2021 | 97,69 |
| Brazil/RS-LMM48516/2021 | 100,00 |
| Brazil/RS-LMM51229/2021 | 99,99 |
| Brazil/RS-LMM54058/2021 | 100,00 |
| Brazil/RS-LMM61916/2021 | 98,70 |
| Brazil/RS-LMM61920/2021 | 98,56 |
| Brazil/RS-LMM61936/2021 | 99,63 |
| Brazil/RS-LMM61938/2021 | 99,59 |
| Brazil/RS-LMM61940/2021 | 99,01 |
| Brazil/RS-LMM61946/2021 | 99,51 |
| Brazil/RS-LMM61949/2021 | 99,96 |
| Brazil/RS-LMM61957/2021 | 99,82 |
| Brazil/RS-LMM61975/2021 | 99,50 |
| Brazil/RS-LMM61976/2021 | 99,40 |
| Brazil/RS-LMM61979/2021 | 99,04 |
| Brazil/SP-IB 117455/2021 | 99,42 |

**Delta**

| sample | coverage breadth (%) |
| --- | --- |
| Argentina/PAIS-G0553/2021 | 98,70 |
| Brazil/PA-ITV-3223/2021 | 99,45 |
| Brazil/RS-LMM62262/2021 | 97,81 |
| Brazil/RS-LMM67349/2021 | 98,36 |
| Brazil/SP-IAL-6731/2021 | 99,33 |
| Brazil/SP-IB_136882/2021 | 97,75 |
| Paraguay/385066/2021 | 95,37 |

**Omicron**

| sample | coverage breadth |
| --- | --- |
| Brazil/MS-LabImuno-220102044533/2022 | 99,93 |
| Brazil/PA-LACENPA-150333209/2022 | 98,95 |
| Brazil/PA-LACENPA-150334384/2022 | 96,77 |
| Brazil/PE-IMTSP-CD33188/2022 | 99,99 |
| Brazil/PR-IMTSP-CD32100/2022 | 100,00 |
| Brazil/RS-LMM72488/2022 | 100,00 |
| Brazil/RS-LMM72559/2022 | 100,00 |
| Brazil/RS-LMM72710/2022 | 99,97 |
| Brazil/SP-IB CEVC 2200108/2022 | 99,35 |
| USA/UT-UPHL-220624610165/2022 | 90,55 |
| USA/UT-UPHL-220708552804/2022 | 86,70 |
| USA/UT-UPHL-220718710062/2022 | 85,66 |
| USA/UT-UPHL-220722933505/2022 | 92,67 |
| USA/UT-UPHL-220816803566/2022 | 93,77 |
| USA/UT-UPHL-220822895779/2022 | 88,15 |
| USA/UT-UPHL-220909951176/2022 | 98,50 |
